# Sex difference in innate inflammatory response and macrophage polarization in *Streptococcus agalactiae*-induced pneumonia and potential role of microRNA-223-3p

**DOI:** 10.1038/s41598-022-21587-5

**Published:** 2022-10-12

**Authors:** Maud Deny, Luis Alexis Arroba Nuñez, Marta Romano, Olivier Denis, Georges Casimir, Mustapha Chamekh

**Affiliations:** 1 Inflammation Unit, Laboratory of Pediatric Research, Faculty of Medicine, Université Libre de Bruxelles, Brussels, Belgium; 2 ULB Center for Research in Immunology (U-CRI), Brussels, Belgium; 3 Immune Response Service, Sciensano, Brussels, Belgium; 4 Queen Fabiola University Children’s Hospital, Brussels, Belgium

**Keywords:** Antimicrobial responses, Inflammation, Innate immune cells, miRNAs

## Abstract

While number of studies have shown that biological sex is a risk factor in the incidence and severity of infection-induced inflammatory diseases, the underlying mechanisms are still poorly understood. In this study, we compared the innate inflammatory response in male and female mice with group B streptococcal (GBS)-induced pneumoniae. Although male and female mice displayed similar bacterial burdens, males exhibited more innate inflammatory cytokines and chemokines and a higher proportion of infiltrating monocytes/macrophages. The analysis of the distribution of macrophage subtypes M1 (pro-inflammatory) versus M2 (anti-inflammatory) yielded a higher M1/M2 ratio in infected males compared with females. Given the importance of the chromosome X-linked microRNA-223-3p (miR-223-3p) in modulating the inflammatory process and macrophage polarization, we investigated its potential contribution in sex bias of GBS-induced innate inflammatory response. Knock-down of miR-223-3p with specific antagomiR resulted in increased inflammatory response and higher M1/M2 ratio following GBS infection. Notably, compared to male mice, we detected higher amount of miR-223-3p in macrophages from females that correlated negatively with M1 phenotype. These results suggest that differential expression of miR-233-3p may impact macrophage polarization, thereby contributing to fine-tune sex differences in inflammatory response.

## Introduction

Group B streptococcus (GBS) or *Streptococcus agalactiae*, is a Gram-positive encapsuled bacteria of the commensal intestinal flora and vaginal cavity. However, it can cause severe pneumonia, meningitis, and sepsis in humans, particularly in newborns and the elderly^1,2^. GBS triggers robust innate inflammatory response that plays a critical role in the control of infection through a direct effect on the pathogen invasion and by shaping subsequent adaptive immune response. Among innate immune cells, phagocytic cells, i.e. neutrophils and monocytes/macrophages, have a prominent role in the inflammatory process and in the elimination of the pathogen. The inflammatory response is generally regarded as a beneficial process for the host in that it promotes an effective immune response against the pathogen. However, it can be detrimental by causing fatal tissue damages when the process goes uncontrolled. Macrophages have a fundamental role not only in host immune defense against pathogens, but also in tissue healing and repair^3,4^*.* Indeed, macrophages exhibit remarkable plasticity and polarize into distinct phenotypes under specific environment stimuli^5^. Macrophages are heterogeneous cells, yet an oversimplification of their polarization allowed their classification into "classically activated" macrophages termed M1 and "alternatively activated" or M2^6^. M1 are associated with a pro-inflammatory phenotype and are essential in the elimination of pathogens, whereas M2 have mainly anti-inflammatory functions and are involved in tissue repair^7^.

There are number of studies indicating that biological sex is a contributing factor to the incidence and progression of several infectious diseases in both human and animal models. In general, males present higher morbidity and mortality than females along the life course^8–10^. In GBS infections, different studies highlighted the influence of biological sex on the incidence and severity of GBS infection, with men having poor prognosis compared to women^11–13^. This is also the case in early life, as studies in infants have shown higher prevalence of GBS sepsis in boys^14^. Although there is accumulating evidence of sex difference in susceptibility to infectious respiratory diseases, investigations on potential mechanisms are still scarce. It is recognized that both the X chromosome-linked sexual genetic architecture and sex hormones are potential contributors^15,16^. Studies have also revealed that prepubertal boys and girls exhibit significant differences in susceptibility to many infectious inflammatory diseases which in support to the essential role of the X chromosome^17,18^. Interestingly, X chromosome is highly rich in gene sequences coding for microRNAs (miRNAs), a group of small (about 22 nucleotides) non-coding RNAs that silence gene expression through binding to the complementary messenger RNA (mRNA) and repressing its translation. Therefore, miRNAs play a cardinal role in the modulation of different biological processes. It is worth noting that several X chromosome-located miRNAs have important functions in immunity and inflammation, which may contribute to sex differences in the immune response and in the dynamic and magnitude of the inflammatory response^19,20^. Among X-linked miRNAs, miR-223, an hematopoietic cell-derived miRNA, plays an important role in the negative regulation of innate immunity^21,22^. Currently, it remains elusive whether, and if so how, miRNAs could contribute to sex bias of the inflammatory response triggered during infection.

We have previously shown in male mice that GBS lung infection results in a rapid expression of miR-223-3p during the first hours of infection, mainly by lung infiltrating neutrophils. We also showed that the accumulation of miR-223-3p in the lung tissue likely contributes to compensatory mechanisms that tend to limit an excessive acute innate inflammation and to restore the immune homeostasis^23^. In this study, we ask whether males and females exhibit contrasting degree of the innate inflammatory response during GBS infection and investigate the potential contribution of X chromosome-linked miR-223-3p in this process.

## Results

### Male and female mice displayed comparable bacterial load during lung infection by a sublethal dose of GBS

**Figure 1:**
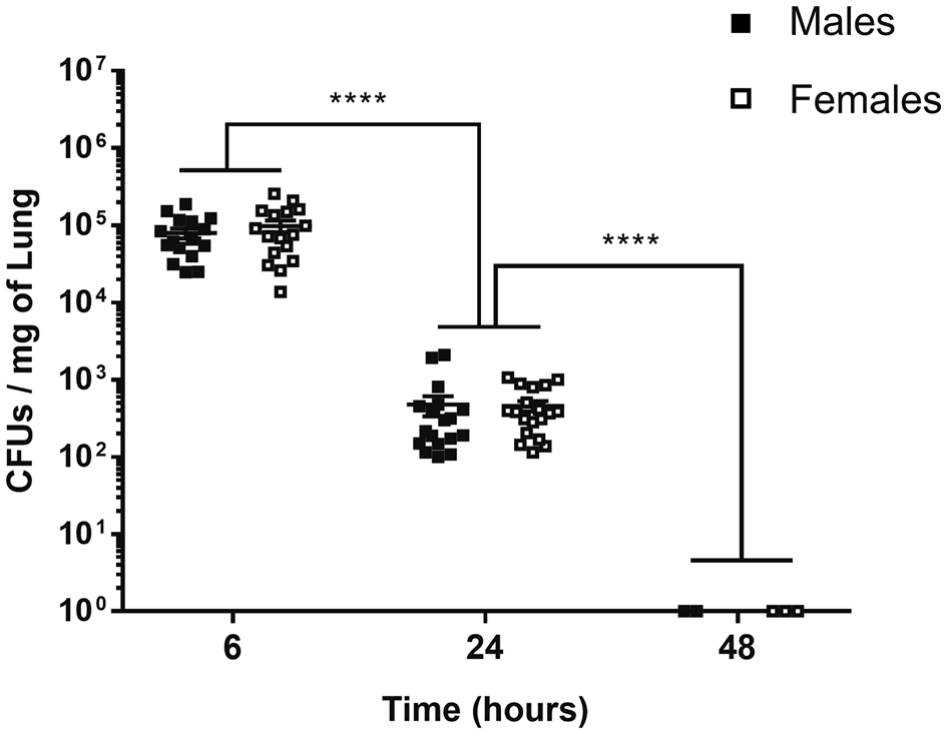
Bacterial count in mice lungs (detection limit: 750 CFUs). C57BL/6 male (black) and female (white) mice were infected intranasally with a sublethal dose (± 10^8^ CFUs) of GBS. ****p < 0.0001 (Mann–Whitney *U* test). Data are representative of three pooled experiments.

To investigate sex difference in innate inflammatory response, we used a mouse model of GBS-induced pneumonia. Two groups of male and female mice were inoculated intranasally with a sub-lethal dose (± 10^8^ CFUs) of bacteria, and the bacterial burden in the lungs was examined for 48 h. As shown in Fig. 1, there was no difference in the bacterial load between males and females and both groups of mice cleared the bacteria within 48 h. Infected male and female mice exhibited comparable weight loss and as expected, no mortality was observed (data not shown). These data indicate that a similar efficiency of bacterial clearance is observed in male and female mice upon infection with a sublethal dose of GBS.

### Higher level of monocytes/macrophages in male mice compared to female mice after intranasal GBS infection

**Figure 2:**
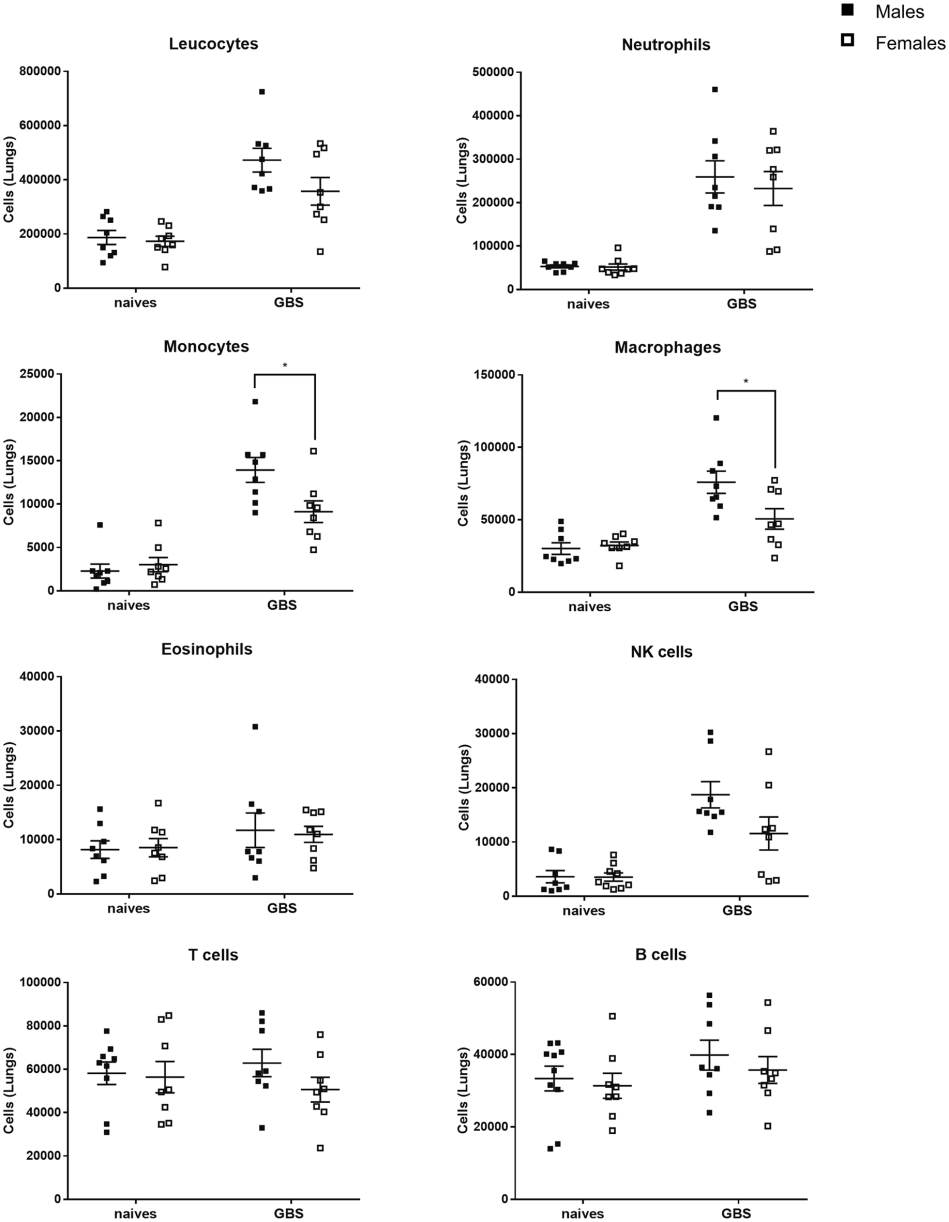
Sex-difference of immune cells in the lungs of naive and GBS-infected mice. Flow cytometry was performed before or after 24 h of GBS infection in male (black) and female (white) mice (n = 8 for each group) using specific antibodies. Levels of leucocytes (CD45 +), neutrophils (CD45+, CD11b+, MHCII−, CD24+, Ly6G+, Siglec F−), eosinophils (CD45+, CD11b+, MHCII−, CD24+, Ly6G−, Siglec F+), monocytes (CD45+, MHCII−, CD11b+, CD64+), macrophages (CD45+, MHCII+, CD64+, CD24−), T cells (CD45+, CD11c−, CD11b−, MHCII−, CD24−), B cells (CD45+, CD11c−, CD11b−, MHCII+, CD24+) and NK cells (CD45+, MHCII−, CD64−, CD11b±) are shown. *p < 0.05 (Mann–Whitney *U* test). Data are representative of two independent experiments.

We then examined lung-infiltrating immune cells by flow cytometry at 24 h post-infection (pi), at which time the innate immune-infiltrating cells culminate under GBS infection^23^ (Fig. 2). The gating strategy used to differentiate the immune cells is shown in Figs. S1 and S2. As expected, significant and massive recruitment of neutrophils (CD45+, CD11b+, MHCII, CD24+, Ly6G+, Siglec F−) and monocytes/macrophages was observed in the lungs of infected mice compared to naive mice. Interestingly, while neutrophils were found at similar counts in both sexes, the number of monocytes (CD45+, MHCII−, CD11b+, CD64+) and macrophages (CD45+, MHCII+, CD64+, CD24−) was significantly higher in males compared to females. We also observed an increase in Natural Killer (NK) cells (CD45+, MHCII−, CD64−, CD11b±) in infected mice compared to naive mice, but no difference was seen between males and females. NK cells are best known for their role in the killing of virus-infected cells. However, they may also play a role in immune responses to bacterial pathogens^24^. No significant difference was observed between naive and infected mice, as well as between male and female mice, regarding the proportion of eosinophils (CD45+, CD11b+, MHCII−, CD24+, Ly6G−, Siglec F+), T cells (CD45+, CD11c−, CD11b−, MHCII−, CD24) and B cells (CD45+, CD11c−, CD11b−, MHCII+, CD24+).

Furthermore, these observations were confirmed by microscopic evaluation of leukocyte cells in bronchoalveolar lavages (BAL) from uninfected and infected mice (Fig. S3). No sex difference in cell counts was observed in the naive state, but after GBS lung infection, we found a significantly higher level of mononuclear phagocytes in the BAL of male mice compared with female mice (Fig. S3).

### Male mice exhibit a marked production of pro-inflammatory cytokines and chemokines compared to female mice following GBS infection

**Figure 3:**
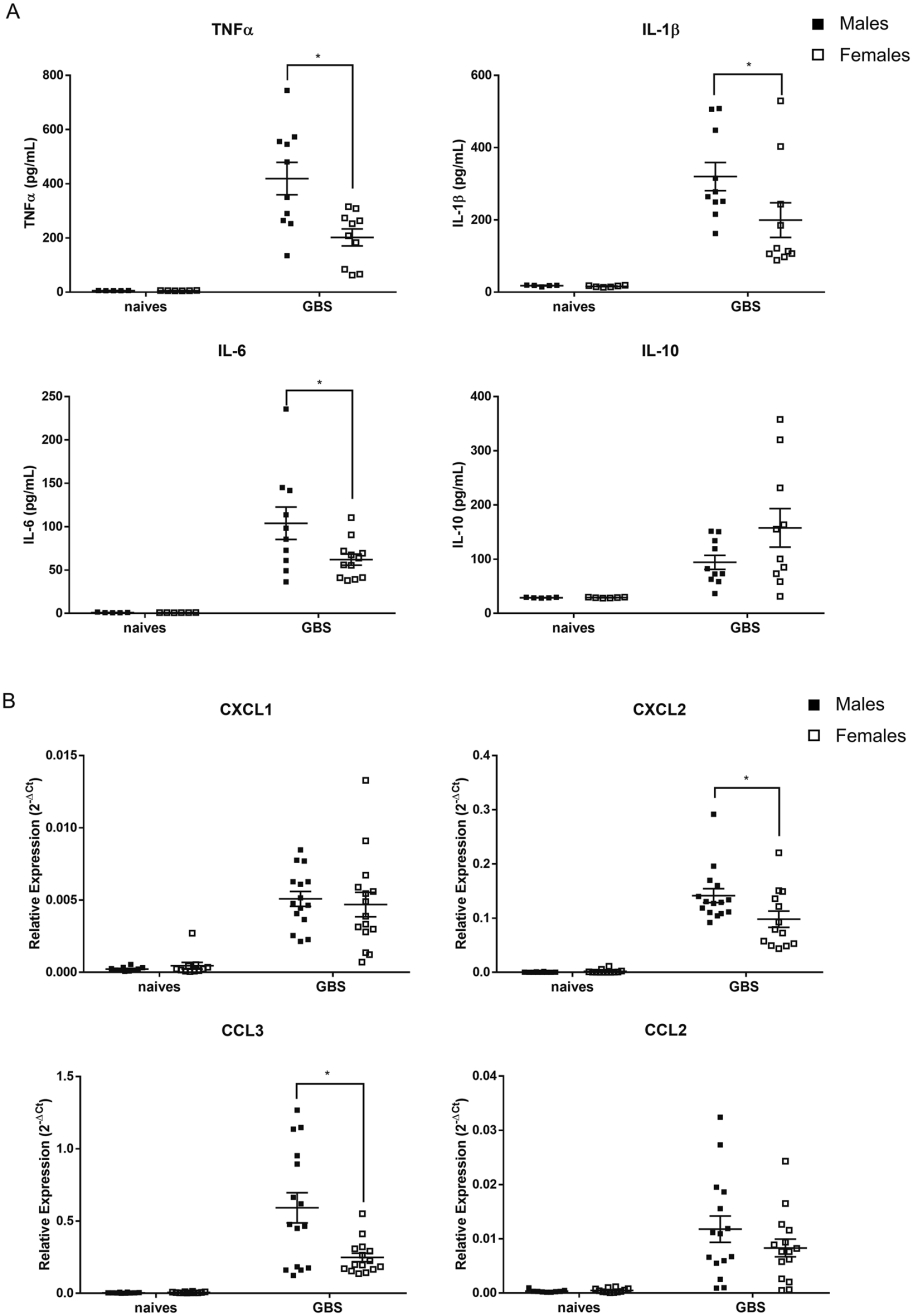
Sex-based assessment of primary cytokine production and chemokines expression in the lungs of GBS-infected mice (24 h pi). (**A**) C57BL/6 male (n = 10) and female (n = 10) mice were infected intranasally with ± 10^8^ CFUs of GBS. TNFα, IL-1β, IL-6 and IL-10 were measured 24 h pi, in suspension fluids of pulmonary tissue by ELISA. For naive mice, data were below the limit of detection in all samples. Extrapolated results are shown. *p < 0.05 (Mann–Whitney *U* test). Data are representative of those from two independent experiments. (**B**) Sex-based assessment of chemokines expression in the lungs of GBS-infected mice. C57BL/6 male (n = 15) and female (n = 15) mice were infected intranasally with ± 10^8^ CFUs of GBS. Relative mRNA transcripts of CXCL1, CXCL2, CCL3 and CCL2 were evaluated 24 h pi in the lungs using real-time RT-PCR. The values were normalized with two internal controls, RPL-19 and β-actin. Relative expression results were compared to those obtained for uninfected male (n = 10) and female (n = 10) control mice. *p < 0.05 (Mann–Whitney *U* test). Data are representative of three independent experiments.

To further evaluate the inflammatory response in the lung tissue between male and female mice after GBS infection, we quantified the local production of primary pro-inflammatory cytokines TNFα, IL-1β and IL-6 by ELISA test. We found a high production of these pro-inflammatory cytokines after GBS infection compared to control mice. Moreover, a significant higher amount of these cytokines was observed in male mice compared to female mice (Fig. 3A). In a similar way, we quantified the production of the anti-inflammatory cytokine IL-10. A trend toward IL-10 overproduction was detected in females compared to males but with no significant difference. In naive mice, the data were below the limit of detection in all samples. We also evaluated by qRT-PCR the mRNA transcripts of key chemokines that have an important role in the recruitment of innate immune cells to the site of infection. As shown in Fig. 3B, the expression of CXCL2 and CCL3 transcripts was significantly higher in infected male compared with infected female mice, in accordance with the elevated level of monocytes recruited to the lungs. However, we did not find a sex difference in the expression of CXCL1 and CCL2 transcripts.

### Distinct M1/M2 macrophages ratio in GBS-infected male and female mice

**Figure 4:**
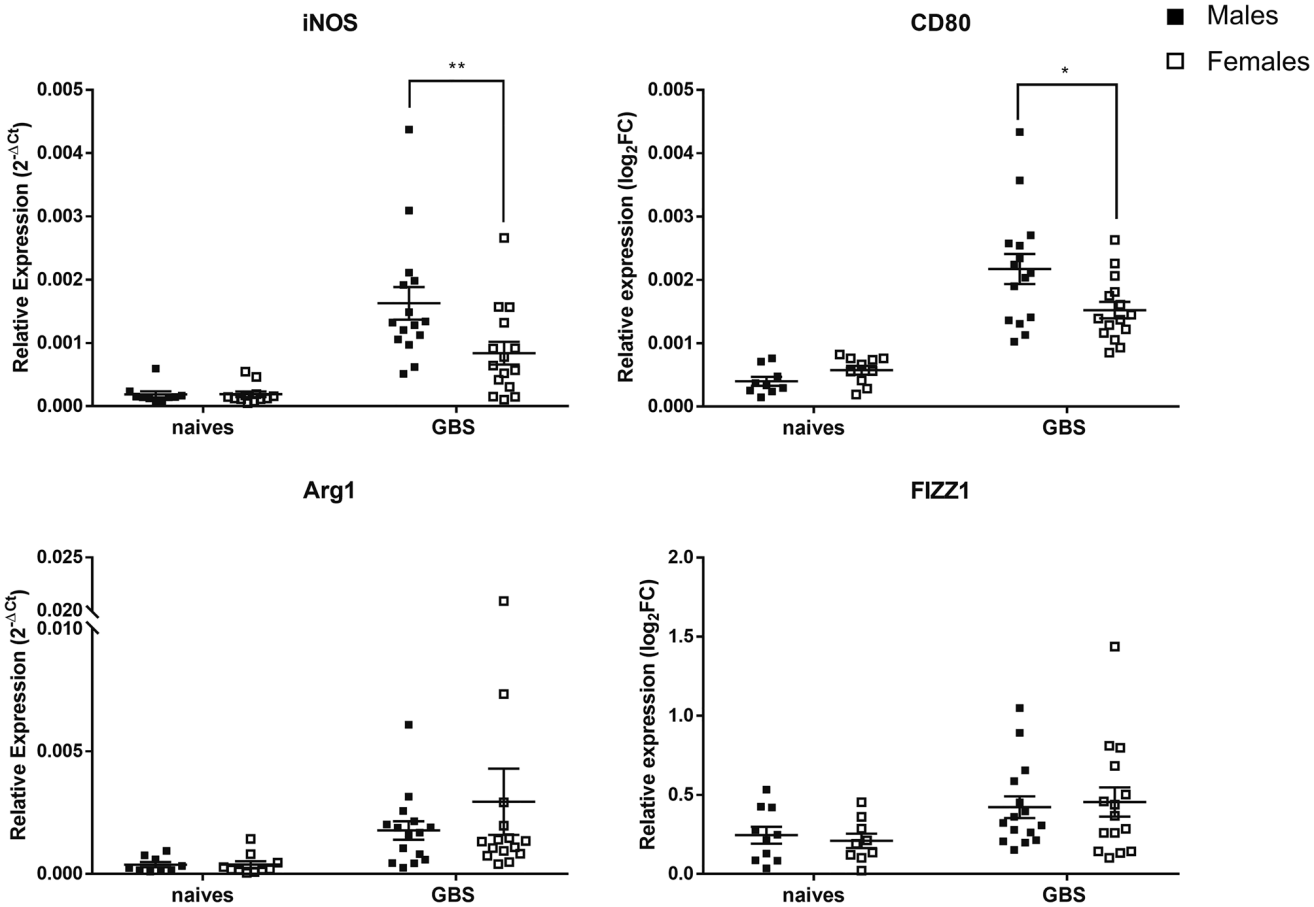
Sex-based assessment of iNOS and CD80 (M1) and, Arg1 and FIZZ1 (M2) expression in the lungs of GBS-infected mice (24 h pi). C57BL/6 male (black) and female (white) mice were infected intranasally with ± 10^8^ CFUs of GBS (n = 15/group). Relative mRNA transcripts of iNOS, CD80, Arg1 and FIZZ1 were evaluated 24 h pi in the lungs using real-time RT-PCR. The values were normalized with two internal controls, RPL-19 and β-actin. Relative expression results were compared to those obtained for uninfected male (n = 10) and female (n = 10) control mice. *p < 0.05, **p < 0.01 (Mann–Whitney *U* test). Data are representative of three independent experiments.

Macrophages can be subtyped into two main phenotypes, classically activated macrophages or M1 which are involved in the pro-inflammatory process and bacterial killing, and alternatively activated macrophages or M2 which contribute to the process of inflammation control and tissue repair. M1 macrophages are characterized by their high capacity to produce inflammatory cytokines, including TNFα, IL-1β, and IL-6, and high amounts of nitric oxide (NO), the product of inducible nitric oxide synthase (iNOS)^25,26^. M2 macrophages are characterized by higher production of the anti-inflammatory IL-10 and ornithine via type I-arginase (Arg1), but lower NO^25,26^. To characterize these two subpopulations in lung tissue, we quantified iNOS and Arg1 expression by qRT-PCR at 24 h pi. We found a significant higher expression of iNOS in male than in female mice (Fig. 4), while the expression of Arg1 was similar between the two sexes (Fig. 4). We also analyzed the co-stimulatory molecule CD80, known to be highly expressed by M1 macrophages^25^, and FIZZ1 (found in inflammatory zone 1, RETNLA), a marker of M2 macrophages^25,27^. Again, we observed a higher expression of CD80 after GBS lung infection in male mice compared to female mice, but no difference was seen in FIZZ1 expression (Fig. 4). These results suggest a predominant M1 type response in male mice, corroborating the more pronounced inflammatory response seen elsewhere (Fig. 3).

**Figure 5:**
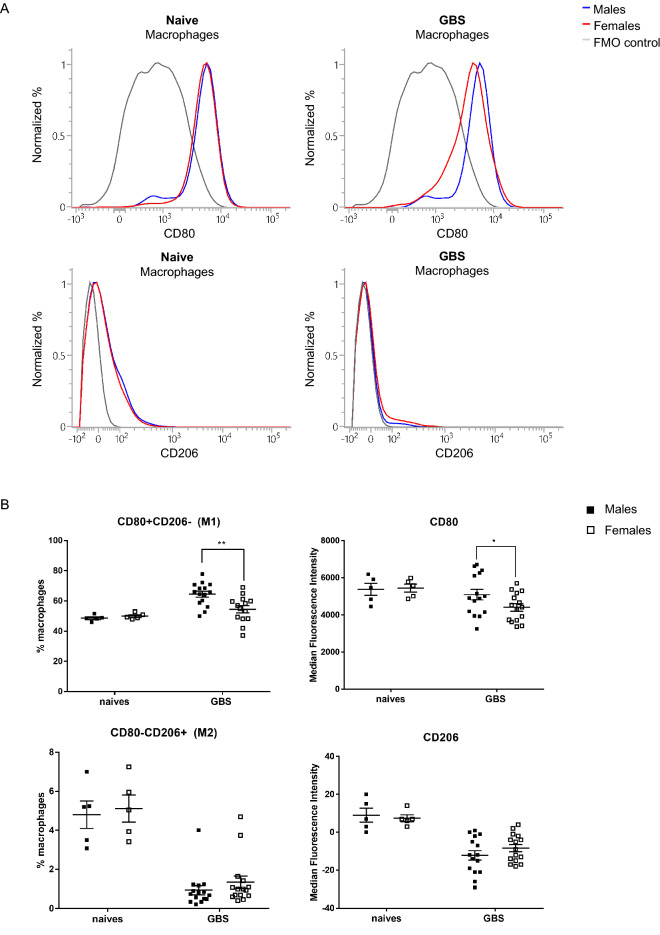
Sex-based analysis of M1 and M2 polarization markers on macrophages at 24 h post GBS lung infection. Male (black) and female (white) C57BL/6 mice were infected intranasally with ± 10^8^ CFU of GBS (n = 15/group). Naive mice were used as controls (n = 5/group). (**A**) Expression levels of CD80 (M1) and CD206 (M2) were compared by flow cytometry on macrophages (CD45+, CD64+, MHCII+) shown in histograms: blue line represent the fluorescent profile of males; red lines represent the fluorescent profile of females and grey lines represent the fluorescent profile for Fluorescence Minus One (FMO) control. (**B**) The % of macrophages positive for surface marker CD80 and negative for CD206 (M1) as well as the % of macrophages positive for surface marker CD206 and negative for CD80 (M2) were determined. Evaluation of the mean fluorescence intensity (MFI) for CD80 and CD206 surface marker expression was also analyzed at the macrophage level. *p < 0.05, **p < 0.01 (Mann–Whitney *U* test). Data are representative of three independent experiments.

To further confirm these results, we immunophenotyped the M1 and M2 macrophage subpopulations by flow cytometry using specific markers, CD80 (M1) and CD206 (M2)^28^. The gating strategy used is shown in Fig. S4. The results showed a significant predominance of M1 phenotype, both in the percentage of positive cells and in the median fluorescence intensity (MFI) in male mice compared to female mice (Fig. 5). In contrast, no difference between male and female mice was observed in the percentage of CD206-positive cells (M2 phenotype) or in CD206 MFI. These data support the imbalance in the M1 versus M2 polarization between male and female mice, with a higher ratio of M1/M2 macrophages in GBS-infected male mice.

### Transient inhibition of miR-223-3p expression results in increase of the proinflammatory profile and CD80 transcript expression upon GBS lung infection

**Figure 6:**
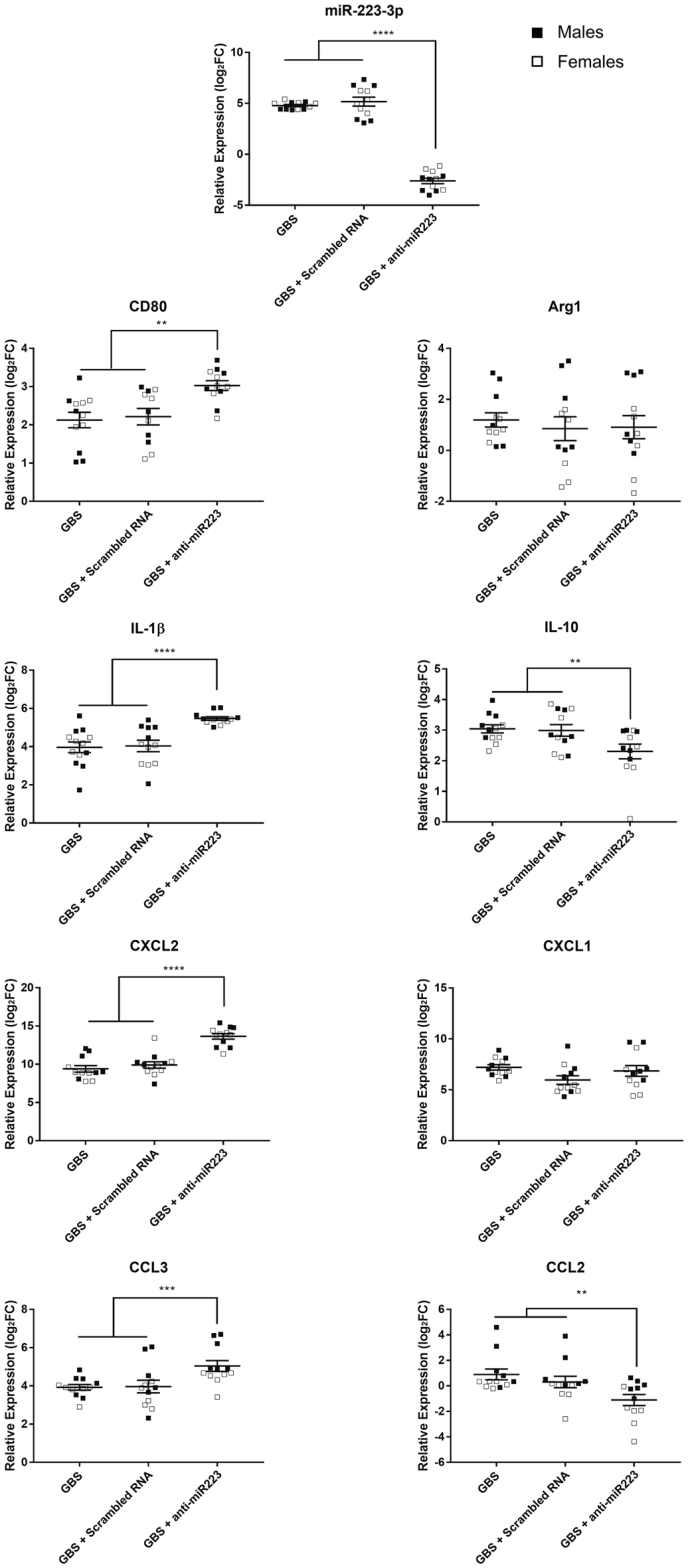
Impact of transient inhibition of miR-223-3p expression by an antagomiR in CD80 and Arg1 as well as pro- and anti-inflammatory cytokines in lung tissue of GBS infected-mice. C57BL/6 male and female mice (n = 6/group) received intranasal injection of GBS alone (± 10^8^ CFUs), or 50 µg/50 µL antagomiR-223, or 50 µg/50 µL scrambled RNA, 30 min before GBS infection and checked for changes in mRNAs of interest 6 h pi. The relative expression of miR-223-3p was evaluated in the lungs by real-time RT-qPCR. The values were normalized with two internal controls, snoRNA-202 and snoRNA-234. The relative expression of mRNAs CD80, Arg1, IL-1β, IL-10, CXCL2, CCL3, CCL2 and CXCL1 was evaluated in the lungs by real-time RT-qPCR. The values were normalized with two internal controls, RPL-19 and β-actin. The fold change values (2^−ΔΔCt^) are relative to naive mice matched by sex and age. Data are means ± SD. **p < 0.01, ***p < 0.001, ****p < 0.0001 (Mann–Whitney *U* test). Data are representative of two independent experiments.

miR-223-3p has been described as an important factor contributing to macrophage polarization and therefore impacting M1/M2 balance^29–34^. In a previous study, we reported that GBS-infected mice rapidly produced miR-223-3p in the lung tissue, that peaked at 6 h pi and remained stable in the late phase of infection. This appears to be one of the compensatory mechanisms to limit the excessive inflammatory response and allow the restoration of the immune homeostasis^23^. To evaluate the implication of miR-223-3p in the polarization of macrophages and the regulation of inflammatory response, we performed transient inhibition experiments by intranasal instillation to male and female mice of an antagomiR-223, or anti-scrambled RNA as a control, 30 min before GBS challenge and we measured the level of CD80, Arg1, IL-1β and IL-10 expression within the lungs at 6 h pi, time at which most innate inflammatory parameters reach their peak^23^. As shown in Fig. 6, the expression of miR-223-3p was strongly decreased following the administration of antagomiR-223 in male and female mice. Consequently, the expression levels of CD80 and IL-1β were significantly increased in antagomiR-223-treated mice compared to control mice. We also observed a significant decrease in IL-10 expression in antagomiR-223-treated mice compared to control mice. However, no change was seen in Arg1 expression.

The impact of the inhibition of miR-223-3p on the expression of chemokines was tested as well (Fig. 6). Male and female antagomiR-223-treated mice exhibited a significant increase of the CXCL2 and CCL3 expression compared to controls. This is in line with studies validating these two chemokines as direct targets of miR-223-3p^22^. We also found in male and female antagomiR-223-treated mice a significant decrease in CCL2 expression, but no change in CXCL1 expression compared to control mice. Overall, these data indicate that miR-223-3p is likely involved in the control of the inflammatory response during GBS infection, at least partly, by the control of M1/M2 ratio and key chemokines that are involved in the recruitment of innate inflammatory cells.

### F4/80+ cells from females express a higher X-linked miR-223-3p than those from males

**Figure 7:**
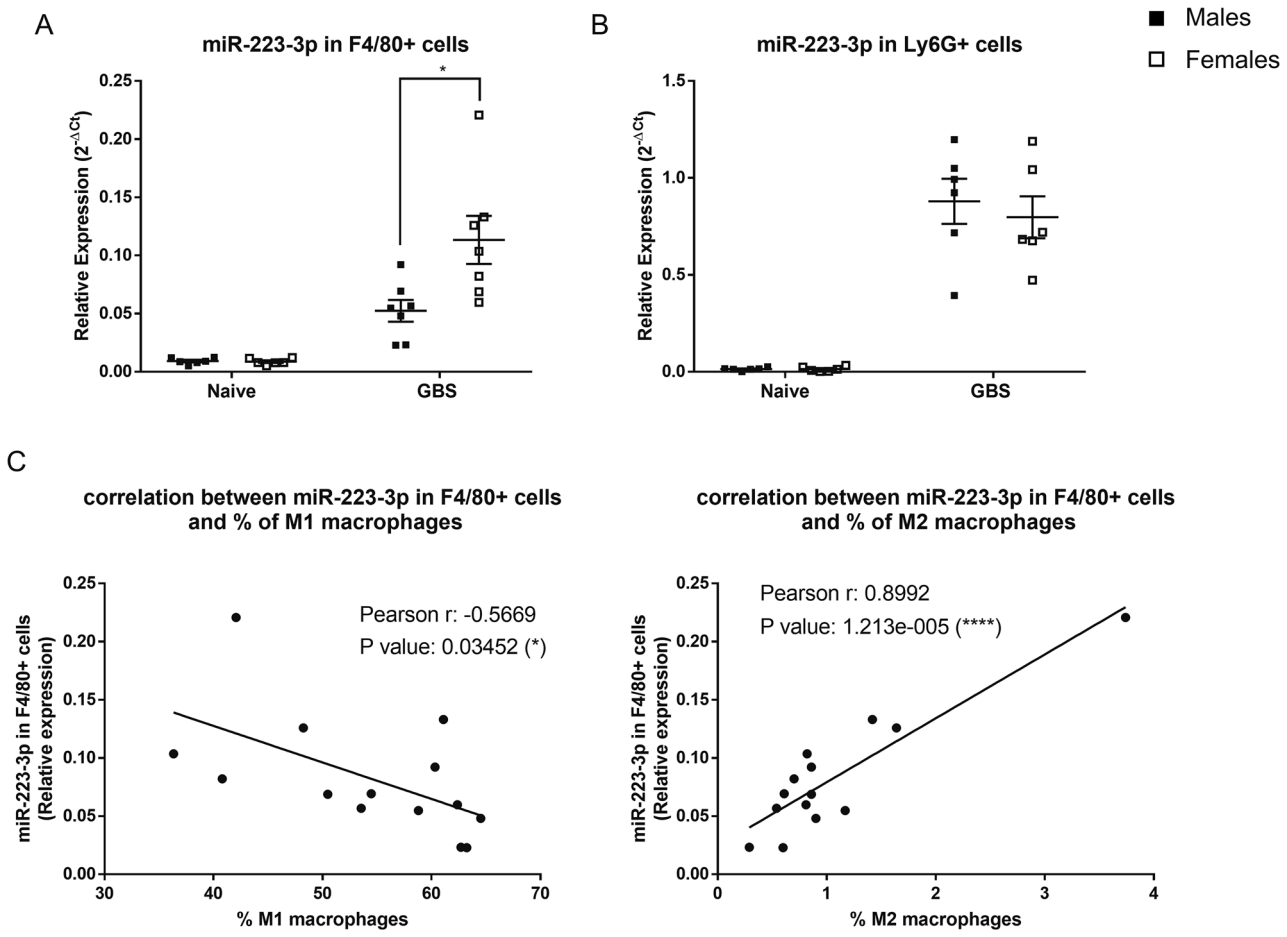
Sex difference in relative miR-223-3p expression in F4/80+ and Ly6G+ cells at 24 h post GBS lung infection. F4/80+ cells and Ly6G+ cells were purified from the lungs of GBS-infected or naive male and female mice (n = 6/group) using F4/80 and Ly6G immunomagnetic antibodies. Comparative analysis of miR-223-3p expression in the F4/80+ (**A**) and in the Ly6G+ (**B**) cell populations was evaluated by real-time qPCR. The values were normalized with two internal controls, snoRNA-202 and snoRNA-234. *p < 0.05 (Mann–Whitney *U* test). Data are representative of two independent experiments. The percentage of CD80+ and CD206+ macrophages were evaluated by flow cytometry with the gating strategy shown in Fig. S4. Correlation between the miR-223-3p expressed by the F4/80+ cells and the percentage of M1 or M2 macrophages (**C**). *p < 0.05, ****p < 0.0001 (Pearson χ^2^ test).

Given the contribution of miR-223-3p in the polarization of macrophages^29–34^, we hypothesized that sex difference in the M1/M2 ratio seen in GBS-induced pneumonia, could be linked to a differential expression of the miR-223-3p between male and female mice. To address this question, we performed purification of lung macrophage using immune bead isolation of F4/80+ cells before and after GBS infection in mice. Interestingly, we found significantly higher expression of miR-223-3p in the F4/80+ cell fraction in female mice compared with male mice upon GBS infection (Fig. 7A). Previously, we have shown that neutrophils elicited during GBS infection are important cellular source of miR-223^23^. Thus, we also compared the expression level of miR-223-3p in Ly6G+ neutrophil cells between GBS-infected male and female mice, but we did not find a significant sex difference (Fig. 7B). To see whether this higher expression of miR-223-3p in macrophages from female mice was associated with a decrease in the M1/M2 ratio, we analyzed the correlation between miR-223-3p expression and the percentage of CD80+ macrophages as well as the percentage of CD206+ macrophages (Fig. 7C). We observed that miR-223-3p expression correlated positively with the percentage of CD206+ macrophages (M2) and negatively with the percentage of CD80+ macrophages (M1). All together, these data suggest that higher expression of miR-223-3p in female lung macrophages likely contributes to the decrease in the M1/M2 ratio and thus promotes more efficient regulation of the innate inflammatory response compared to male mice during GBS lung disease.

## Discussion

It is well documented that biological sex is a contributing factor to the incidence and progression of a number of diseases, including bacteria-induced acute respiratory diseases^8–10,35,36^. Regarding GBS, human studies reported that the incidence and severity of infection is higher in men than women^11–13^. The severity of GBS infection is mainly attributed to the higher inflammatory potential of invading bacteria. However, the potential mechanisms underlying the distinct outcomes in males and females have not yet been analyzed. To our knowledge, this is the first GBS experimental study to compare the innate inflammatory response in males and females, including the characterization of innate infiltrating cells, and to address whether miRNAs could play a role.

We showed that although male and female mice infected with a sublethal dose of GBS have a comparable efficiency in clearing the bacteria from the lung, male mice exhibited higher local production of pro-inflammatory cytokines TNFα, IL-1β and IL-6 and chemokines CXCL2 and CCL3. It is generally agreed that clearance of invasive pathogens relies on the inflammatory and protective immune response, the magnitude of which must be tightly controlled. However, in most cases, the strong innate inflammatory response triggered in the early phase of infection in males seems to be deleterious. It has been shown in different acute infectious models that the more severe symptoms usually observed in males correlate with a higher level of innate inflammatory response, but not with the overall number of invading pathogens. The same observations were recently reported in SARS-Cov-2 infection, where more severe symptoms and higher mortality were observed in males compared with females^37–39^. Males exhibited a similar viral load to females despite their higher levels of innate inflammatory cytokines and chemokines^37^. These observations are consistent with the hypothesis that the magnitude of the inflammatory response, rather than the pathogen load, may play a prominent role in infectious pathogenesis. It remains unclear whether the innate cells from males and females display different phagocytic activity. It would be interesting to investigate this question in the GBS-induced inflammatory context.

Differences in the inflammatory cytokine response between male and female mice have been reported in *Streptococcus pneumoniae* infection model^40^. Our study on GBS infection model goes further by analyzing the innate infiltrating cells. Interestingly, we found a higher M1/M2 macrophages ratio in infected male mice compared to infected female mice, corroborating the more prominent inflammatory profile observed in males. Macrophages have a fundamental role not only in the host immune defense against pathogens and in the inflammatory process, but also in healing and repairing tissue^3,4^*.* Indeed, macrophages exhibit remarkable plasticity under specific stimuli of the microenvironment and polarize into cells with distinct phenotypes that differ in their gene expression profile and cytokine production pattern^5^. M1 “classically activated” and M2 “alternatively activated” macrophages refer to the two extremes of a spectrum of a wide variety of macrophage polarization states^6^. M1 macrophages have a pro-inflammatory phenotype and are essential in the elimination of pathogens, whereas M2 macrophages have mainly anti-inflammatory functions and are involved in tissue repair^7^. It is noteworthy that our data are consistent with those reported in a Coxsackievirus-induced myocarditis model showing that male mice expressed higher levels of classically activated M1 macrophages infiltrating the myocardium and exhibited more severe inflammatory symptoms compared to females^41^. Therefore, it is likely that a trend towards higher M1/M2 ratio is a contributing factor to the sex difference in the magnitude of the infection-induced inflammatory response.

With the aim to study the potential contribution of chromosome X-linked miRNAs in sex bias of GBS-induced innate inflammatory response, we focused on miR-223-3p given its prominent role in modulating inflammation and its impact on macrophage polarization^29–34^. We previously showed in GBS-infected mice that specific inhibition of miR-223-3p by an antagomiR resulted in a significant increase of CXCL2^23^. In this study, we extended our analysis to additional inflammatory markers and found a significant overexpression of CCL3, IL-1β as well as a decrease in the expression of IL-10 in antagomiR-223-3p treated male mice. Moreover, we observed a significant increase in CD80 expression, suggesting the predominance of M1 phenotype. Conversely, miR-223-3p knock-down leaded to a significant decrease in CCL2 expression. Interestingly, CCL2 was reported to be preferentially expressed by M2 macrophages^42^. These observations are consistent with the key role attributed to miR-223-3p as a negative regulator of the innate immune response by targeting NLRP3 and STAT3^43–46^ and by the control of myeloid cell recruitment by targeting CXCL2, CCL3^22^.

We and others have previously shown that neutrophils are an important cellular source of miR-223-3p^22,23,43^, but other immune cells such as macrophages can also produce this miRNA in detectable amounts^47,48^. When comparing the level of expression of miR-223-3p in neutrophil cell population from infected male and female mice, we did not observe significant difference. However, we found a higher miR-223-3p expression in macrophages from female mice compared to male mice. It is reasonable to argue that this differential expression between males and females may impact M1/M2 ratio given the role of miR-223-3p in macrophage polarization^29–34^. In support to this, the overexpression of miR-223-3p in female lung macrophages correlates positively with the percentage of CD206+ macrophages (M2), and negatively with the percentage of CD80+ macrophages (M1). The association of macrophage polarization and their phagocytic activity has been proposed^49^. With respect to our GBS infection model, it would be interesting to evaluate whether macrophages from males and females have a differential degree of bacterial phagocytosis and, if so, to what extent this could be related to miR-223-3p expression.

The higher level of miR-223-3p expression in female macrophages may be explained by gene silencing escape. It is worth noting that one of the two X chromosomes is naturally inactivated in females to ensure the compensation dosage between the two sexes^50^. Yet, it has been shown that certain X-chromosome-linked gene sequences may escape the inactivation process, which could lead to their hyper-production in females^51,52^. Whether X-linked miR-223 is subjected to silencing escape, which would favor its over-expression in females under inflammatory settings, is an interesting way to explore. It should also be noted that the analysis of the distribution of miR-223-3p expression in males and females was performed on highly purified cell fractions. It is therefore possible that other cells producing miR-223-3p may participate in the modulatory process. Another potential mechanism by which miR-223-3p may impact macrophage polarization is the communication between immune cells. A recent study on liver inflammation model reported that neutrophils could induce macrophage polarization through the transfer of neutrophil-extracellular vesicles containing miR-223-3p, hence leading to the attenuation of the inflammatory process^53^. Similarly, another study highlighted that in the lungs, intracellular delivery of miR-223-3p via microvesicles suppressed macrophage activation and pulmonary inflammation through inhibition of NLRP3 inflammasome activation^54^. Although we did not observe sex difference in the expression level of miR-223-3p in neutrophils, we cannot exclude a dynamic process in vivo that allows the uptake of microRNA-containing neutrophil-derived extracellular vesicles by macrophages. Our study provides evidence for the potential contribution of miR-223-3p in fine-tuning sexual dimorphism of the inflammatory response, at least partly through macrophage polarization. Further studies are warranted to pinpoint the molecular mechanism at work.

## Materials and methods

### Bacterial strains

The clinical isolate of Streptococcus agalactiae (COH1; serotype III) was kindly provided by Victor Nizet, University of California San Diego, La Jolla, CA. Bacteria were grown in Todd Hewitt broth (THB) with 15 mg/mL nalidixic acid at 37 °C without shaking, until exponential phase. Enumeration of CFUs (colony forming units) on Todd Hewitt Agar (THA) media plates with 15 mg/mL nalidixic acid was then performed, and a stock of aliquots of known concentration was stored at − 80 °C with 50% glycerol. At the time of use, aliquots of bacteria were thawed and centrifuged (5000 rpm for 5 min) and then resuspended in PBS at the desired concentration for inoculation (± 10^8^/100 µL). To verify the inoculum dose, the CFUs count of each aliquot was checked once more (representative data are shown in Table S1).

### Pulmonary infection mice

All experiments with mice were approved by the Institutional Animal Welfare Committee of Sciensano (approval reference numbers 20180125-01 and 20210113-01). The experiments were carried out in accordance with the current European legislation (Directive 86/609/CEE), in respect with the Belgian law, and the study is in accordance with ARRIVE guidelines (https://arriveguidelines.org). After anesthesia with 4% isoflurane, C57BL/6 male and female mice (± 4 weeks old) were inoculated intranasally with the sublethal dose of ± 10^8^ CFUs/100 µL GBS. To evaluate GBS virulence, disease signs and weight loss were monitored and bacterial levels in the lungs were measured.

### Analysis of the infiltrated cell populations

Lung cell populations were analyzed by flow cytometry and quantitative microscopic assessment of leukocyte cells in bronchoalveolar lavages (BAL). For the flow cytometry analyze, the mice were sacrificed 24 h pi and the lungs were collected and crushed in 5 mL of PBS. 3 mL was processed for 30 min at 35 °C and 5% of CO_2_, in digestion buffer (1 mg/mL of collagenase A and 0.1 mg/mL of DNase I in Hanks balanced salt solution [HBSS] with 5% fetal bovine serum). Single-cell suspensions were stained with a LIVE/DEAD fixable aqua dead cell stain kit (Invitrogen) according to the manufacturer’s instructions. Cells were incubated in the dark with a panel of specific antibodies for 30 min on ice. Panel 1 included I-A/I-E fluorescein isothiocyanate (FITC), CD45 phycoerythrin (PE), CD11c PE-Cy^TM^5.5, CD11b PE-Cy^TM^7, CD24 APCeFluor 780 (Thermo Fisher Scientific), and CD64 BV421 (BioLegend). Panel 2 included I-A/I-E FITC, CD45 PE, Ly6G peridinin chlorophyll protein (PerCP)-Cy^TM^5.5, CD11b PE-Cy^TM^7 (Thermo Fisher Scientific), Siglec F APC-Cy^TM^7, and CD24 BV421 (BD Biosciences). After staining, cells were washed and fixed with 4% paraformaldehyde. Data were acquired with a BD FACSVerse flow cytometer and analyzed with BD FACSuite flow cytometry software. Gates were determined by fluorescence minus one (FMO) control.

For the microscopic analysis, mice were sacrificed 24 h pi and BAL were performed by injecting 800 µL of PBS into the trachea twice with an 18-gauge needle. Cytospins (500 rpm 5 min) were then performed, and the cells were fixed and colored with the Diff-QuiK kit (Medion Dx). At least 600 leukocytes were counted under the microscope.

### Detection of inflammatory cytokines

The quantification of TNFα, IL-1β, IL-6 and IL-10 levels in the lungs 24 h pi was performed by ELISA. Briefly, the lungs were collected and homogenized in 5 mL of PBS. 1 mL was centrifuged and the cell-free suspensions were recovered for ELISAs performed with specific kits (Invitrogen; 88-7324-88, 88-7013-88, 88-7064-88, 88-7105-88) according to the manufacturer’s instructions. Plate reading and quantification were performed using the Infinite F50 absorbance microplate reader, and the results were analyzed using the Magellan program.

### RNA expression analysis

24 h after intranasal administration of GBS, mice were sacrificed, and the lungs were collected and crushed in 5 mL of PBS and 1 mL was recovered. After centrifugation, cell pellets were treated with 700 µL of QiaZol lysis reagent (Qiagen) and stored at − 80 °C. Total RNA was extracted using the miRNeasy minikit (Qiagen) following the manufacturer’s instructions. For miRNA expression analysis, reverse transcription was performed with a miScript II RT kit (Qiagen) following the manufacturer’s instructions. For the expression of mRNAs transcripts, reverse transcription was performed with the GoScript reverse transcription system kit (Promega) according to standard protocols. The qRT-PCR was performed with the GoTaq qPCR Master Mix (Promega). For quantification of miRNAs by qRT-PCR, two internal miRNA controls were used: two small nucleolar RNAs (snoRNAs), snoRNA-202 and snoRNA-234 that have been previously validated as stable housekeeping genes^55^. For mRNA expression analysis, two housekeeping genes (beta-actin, and RPL-19) were used as internal controls. A no-template control and no-reverse transcriptase control were performed, and no amplicon was detected. The specificity of each primer pairs was further verified by controlling the melt curve profile. The primers used in this study are listed in Table 1. Relative quantification of miRNA and mRNA expression was calculated using the threshold cycle (2^−ΔΔCT^) method. The fold change values were relative to those from naive mice.

**Table 1:** Oligonucleotide primers used for miRNA and mRNA qRT-PCR analysis.

Gene	Sequence (5′ → 3′)	
	Forward primer	Reverse primer
miR-223-3p	TGT-CAG-TTT-GTC-AAA-TAC-CCC-A	miScript Universal Primer (Qiagen)
snoRNA-202	AGT-ACT-TTT-GAA-CCC-TTT-TCC-A	miScript Universal Primer (Qiagen)
snoRNA-234	TTA-ACA-AAA-ATT-CGT-CAC-TAC-CA	miScript Universal Primer (Qiagen)
CXCL1	CCG-AAG-TCA-TAG-CCA-CAC-TCA-A	GCA-GTC-TGT-CTT-CTT-TCT-CCG-TTA-C
CXCL2	AGA-CAG-AAG-TCA-TAG-CCA-CTC-TCA-AG	CCT-CCT-TTC-CAG-GTC-AGT-TAG-C
CCL3	ACA-GCC-GGA-AGA-TTC-CAC-GCC	CAA-GCC-CCT-GCT-CTA-CAC-GGG
CCL2	AGA-GAG-CCA-GAC-GGA-GGA-AG	GTC-ACA-CTG-GTC-ACT-CCT-AC
IL-1β	GTG-TGG-ATC-CAA-AGC-AAT-AC	GTC-TGC-TCA-TTC-ATG-ACA-AG
IL-10	GGT-GTC-CTT-TCA-ATT-GCT-CTC-AT	TCA-CAA-CTC-TCT-TAG-GAG-CTC-TGA-ACT
CD80	GGC-AGC-CAA-ACT-TCT-CAA	CCC-TGC-CCT-CCT-GGT-TTT-TCT
Arg1	TGA-ACA-CGG-CAG-TGG-CTT-TA	GCA-TTC-ACA-GTC-ACT-TAG-GTG-GTT-TA
iNOS	GGA-GCA-GGT-GGA-AGA-CTA-TTT-CTT	CAT-GAT-AAC-GTT-TCT-GGC-TCT-TGA
Fizz1	CCC-AGG-ATG-CCA-ACT-TTG-AA	GGC-CCA-TCT-GTT-CAT-AGT-CT
RPL-19	GAA-GGT-CAA-AGG-GAA-TGT-GTT-CA	CCT-TGT-CTG-CCT-TCA-GCT-TGT
β-Actin	AAA-TCT-GGC-ACC-ACA-CCT-TC	GGG-GTG-TTG-AAG-GTC-TCA-AA

### Analysis of M1 and M2 macrophages

The polarization of macrophages was analyzed by flow cytometry. 24 h pi, the lungs were harvested and processed for 30 min at 35 °C and 5% of CO_2_, in digestion buffer (1 mg/mL of collagenase A and 0.1 mg/mL of DNase I in Hanks balanced salt solution [HBSS] with 5% fetal bovine serum). Single-cell suspension were stained with a LIVE/DEAD fixable aqua dead cell stain kit (Invitrogen) according to the manufacturer’s instructions. Cells were incubated with a panel of specific antibodies for 30 min on ice in the dark. The panel 3 included I-A/I-E FITC, CD45 PE, CD11c PE-Cy^TM^5.5, CD11b PE-Cy^TM^7, CD64 APC, CD206 APC-Cy^TM^7, and CD80 BV421 (Thermo Fisher Scientific). After staining, cells were washed and fixed with 4% paraformaldehyde. Data were acquired with a BD FACSVerse flow cytometer and analyzed with BD FACSuite flow cytometry software. The gates used to properly interpret the data were determined by fluorescence minus one (FMO) control.

### AntagomiRs administration

AntagomiRs were pruchased from Eurogentec. The sequence of the anti-miR-223-3p is *5*′-mC*mA*mC mUmUmG mGmGmG mUmAmU mUmUmG mAmCmA mAmAmC mUmGmA mCmA*mC* mU*mC*mU-3′*-chol* and the sequence of scrambled RNA is *5*′-mG*mC*mA mAmGmU mCmAmA mUmUmA mCmCmG mUmCmU* mC*mU*mA* mU-3′*-chol*, where m denotes 2-O-methyl-modified phosphoramidities, ∗ denotes phosphorothioate linkages, and *chol* denotes hydroxyprolinol-linked cholesterol. We administered intranasally, 50 μg of anti-miR-223-3p or anti-scrambled RNA suspended in 50 μL of sterile PBS, 30 min before the intranasal GBS infection (10^8^ CFUs). 6 h later, the mice were sacrificed, and the lungs recovered for expression analysis.

### Immunomagnetic purification of Ly6G+ and F4/80+ cells

25.10^5^ lung single cells were incubated with 25 µL of anti-mouse Ly6G biotin 0.5 mg/mL (BioLegend) and 10^7^ lung single cells were incubated with 100 µL of anti-mouse F4/80 biotin 0.5 mg/mL (BioLegend) for 30 min at 4 °C and washed with PBS, 2 mM of EDTA and 0.5% of bovine serum albumin (BSA). After centrifugation, the cell pellet was incubated for 30 min on ice with 5 µL/10^6^ cells of Streptavidin Particle Plus-DM (BD Biosciences). The sample volume was then adjusted to 1 mL with PBS, 2 mM of EDTA and 0.5% BSA and placed in a magnetic rack for 7 min. The supernatant was eliminated, and the labeled cells were recovered in 1 mL of PBS, 2 mM of EDTA and 0.5% BSA and placed again in the magnetic rack. This procedure was repeated four times. After centrifugation, cell pellets were treated with 700 µL of QiaZol lysis reagent (Qiagen) and stored at − 80 °C until use. To determine the purification rate, cells were analyzed by flow cytometry. Cells were stained with a LIVE/DEAD fixable aqua dead cell stain kit (Invitrogen) according to the manufacturer’s instructions. Ly6G+ cell fractions were incubated 30 min on ice with CD45 PE, Ly6G FITC, and CD11b PE-CyTM7 (Thermo Fisher Scientific). The Ly6G+ cell fraction represents 90% of live neutrophils. F4/80+ cell fractions were incubated 30 min on ice with CD45 PE, I-A/I-E FITC (Thermo Fisher Scientific), and F4/80 BV421 (BD Biosciences). The F4/80+ cell fraction represents 96% of live macrophages.

## Supplementary Information

Supplementary Information.

## Data Availability

Flow cytometry datasets generated during the current study are available in the FlowRepository (http://flowrepository.org), FR-FCM-Z5FL, FR-FCM-Z5FM, FR-FCM-Z5FT. These experiments have been locked for reviewers' access and may be accessed via the following URL: http://flowrepository.org/id/RvFriKu4Q1uZhE0FFhkjSAFBUw2CL75fjIlN7SblfxF7byVdJxfaosmynR9n2aML, http://flowrepository.org/id/RvFr2OqZyhKxLKWgSV0g8q9AXpT2SGpZZH9hRbGc8lBISa34eHhJrj3Vzh6sN5s7, http://flowrepository.org/id/RvFrtpGDTxtwiXnqykYe5KFs7GEn5OgMWbqTD9c0swp8dYeNPMLQMrkQN8K5q4ir. ELISA and qRT-PCR datasets generated and analyzed during the current study are available in the Open Science Framework. These experiments have been locked for reviewers' access and may be accessed via the following URL: https://osf.io/uafbx/?view_only=f000dc0a6b9445478bf60b895bac4165. If you need more details on the data that support the results of this study, please contact Maud Deny (maud.deny@ulb.be).
