# Supplementary material for: Sex difference in innate inflammatory response and macrophage polarization in Streptococcus agalactiae-induced pneumonia and potential role of microRNA-223-3p
Source: Sci Rep. 2022 Oct 12;12:17126. doi: 10.1038/s41598-022-21587-5 (PMC9555696; doi:10.1038/s41598-022-21587-5)
Supplement: Supplementary file 1 — Supplementary Information. [file 41598_2022_21587_MOESM1_ESM.pdf]

Sex difference in innate inflammatory response and macrophage polarization in *Streptococcus agalactiae*-induced pneumonia and potential role of microRNA-223-3p

Maud Deny[1], Luis Alexis Arroba Nuñez[1], Marta Romano[2], Olivier Denis[2], Georges Casimir[1] and Mustapha Chamekh[1]*

[1] Inflammation Unit, Laboratory of Pediatric Research, Faculty of medicine, Free university of Brussels (Université libre de Bruxelles)

[2] Immune Response Service, Sciensano, Brussels, Belgium.

* Corresponding author: mchamekh@ulb.ac.be

Supplementary data

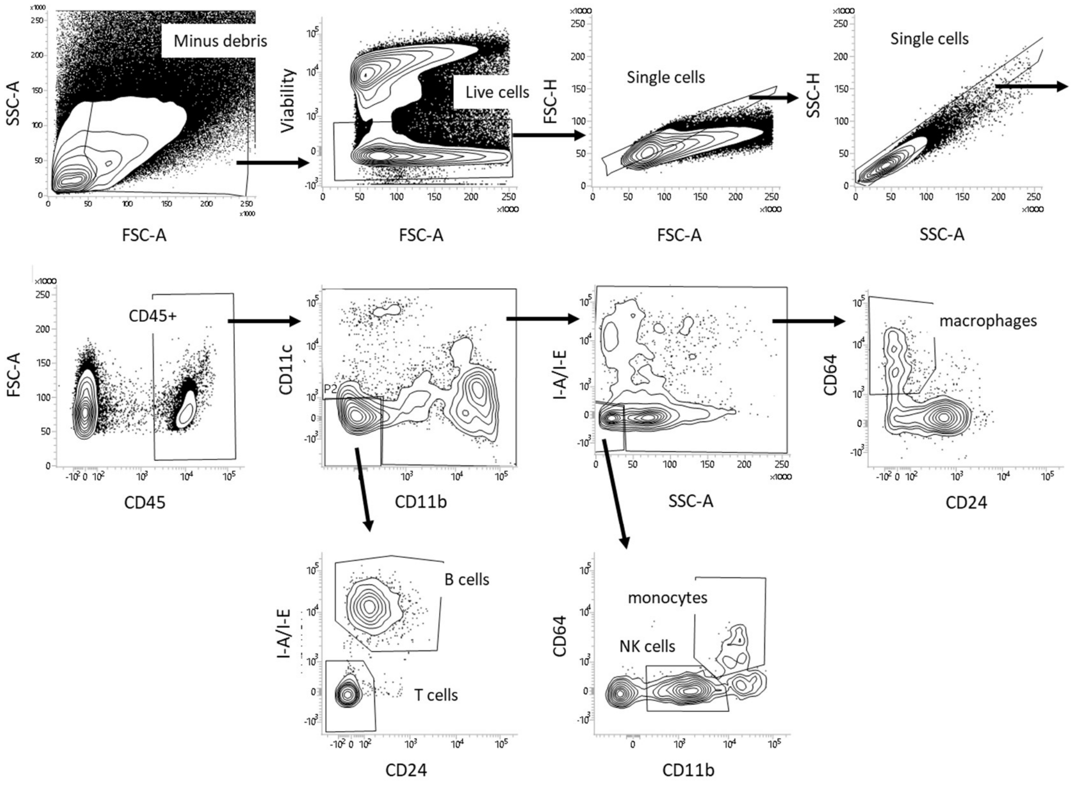

Figure S1. Gating strategy followed in flow cytometry analysis of monocytes, macrophages, B cells, T cells and NK cells in the lungs of naive and GBS-infected mice.

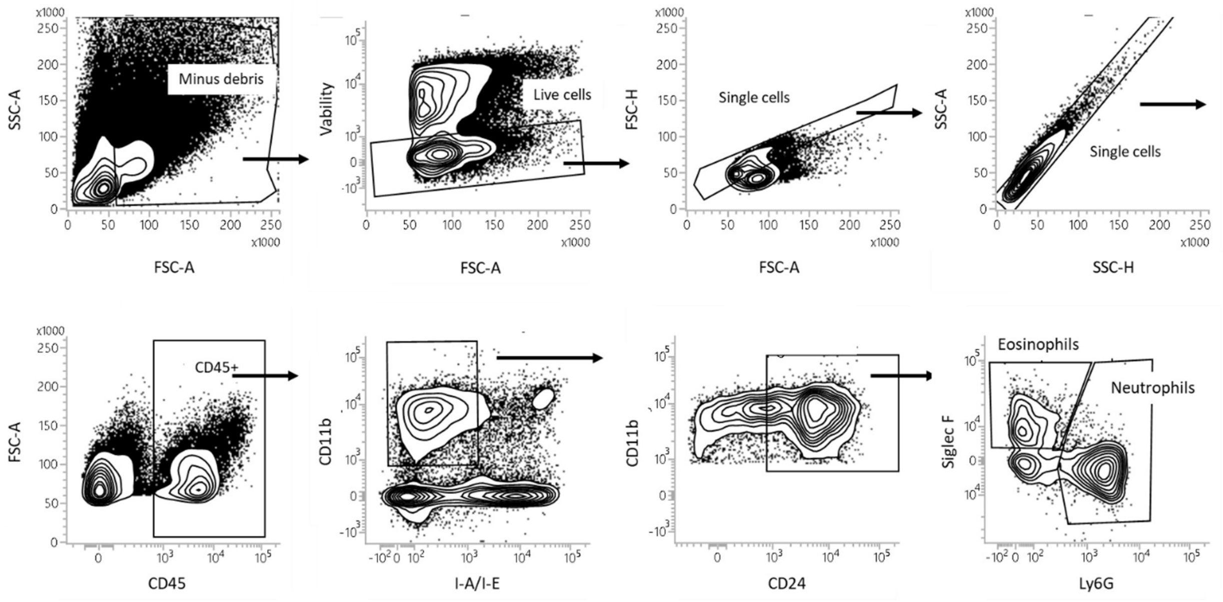

Figure S2. Gating strategy followed in flow cytometry analysis of neutrophils and eosinophils in the lungs of naive and GBS-infected mice.

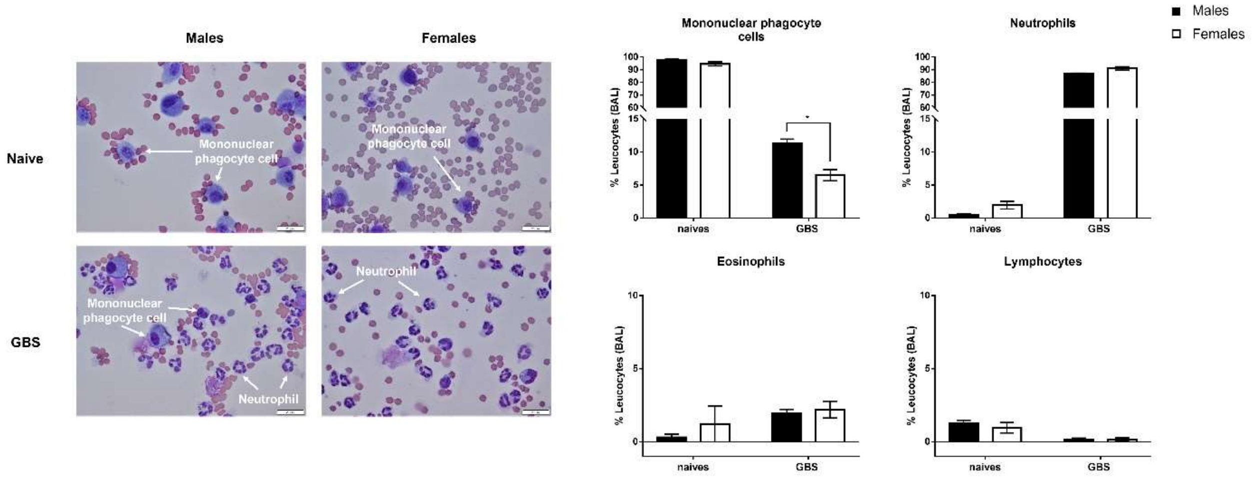

Figure S3. Sex difference in leukocyte cells in bronchoalveolar lavage (BAL) in naive and GBS-infected mice. C57BL/6 male (black) and female (white) mice were infected intranasally with ±10^8 CFUs of GBS. Age-matched naive male and female mice were used as controls (n=5 for each group). 24h pi, BALs were performed, and the recovered cells were fixed and stained with Diff-QuiK staining kit. A minimum of 600 cells were counted by microscopy. Data are means ±SD. *p<0.05 (Mann-Whitney U test).

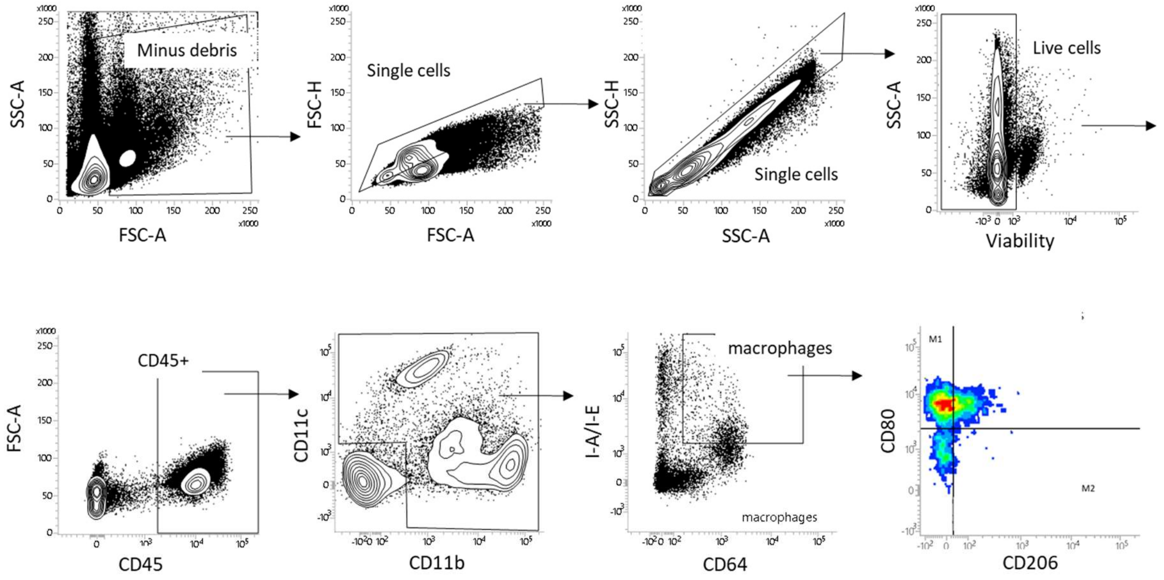

Figure S4. Gating strategy followed in flow cytometry analysis of CD80+CD206- macrophages (M1) and CD80-CD206+ macrophages (M2) in the lungs of naive and GBS-infected mice.

**CFUs count before frozen at -80°C with 50% glycerol**			**CFUs count after thawing and resuspension with sterile PBS at the desired concentration ($1*10^9$/mL).**		
			**Test 1**		
Dilution factors	Mean CFUs count	number of GBS in 1 stock tube	Dilution factors	Mean CFUs count	number of GBS/mL
$6*10^6$	145	$8.17*10^8$	$4*10^6$	235	$9.4*10^8$
$1*10^7$	52	$5.2*10^8$	$4*10^7$	24	$9.6*10^8$
mean		**$6.95*10^8$**	mean		**$9.5*10^8$**
			**Test 2**		
			Dilution factors	Mean CFUs count	number of GBS/mL
			$2*10^7$	75	$1.5*10^9$
			$2*10^8$	8	$1.6*10^9$
			mean		**$1.55*10^9$**

Table S1. Representative data of CFUs (colony forming units) enumeration on Todd Hewitt Agar (THA) media plates with 15mg/mL nalidixic acid, after freezing and thawing and resuspension to the desired concentration.
